# The Multi-Dimensional Role of Vitamin D in the Pathophysiology and Treatment of Diabetic Foot Ulcers: From Molecular Mechanisms to Clinical Translation

**DOI:** 10.3390/ijms26125719

**Published:** 2025-06-14

**Authors:** Weiwei Tang, Shengqiu Chen, Shuxia Zhang, Xingwu Ran

**Affiliations:** 1 Department of Endocrinology and Metabolism, West China Hospital of Sichuan University, Chengdu 610041, China; tangweiwei95@163.com; 2 Innovation Research Center for Diabetic Foot, Diabetic Foot Care Center, West China Hospital of Sichuan University, Chengdu 610041, China; 3 Research Core Facilities, West China Hospital, Sichuan University, Chengdu 610041, China; 4 Center for High Altitude Medicine, West China Hospital, Sichuan University, Chengdu 610041, China

**Keywords:** vitamin D, diabetic foot ulcers, vitamin D receptor, pathophysiology, wound healing, mechanism, clinical translation

## Abstract

Diabetic foot ulcers (DFUs) constitute a severe and debilitating complication of diabetes, imposing a substantial global health burden due to their intricate pathophysiology and impaired wound healing processes. Vitamin D deficiency is highly prevalent among diabetic populations, and accumulating evidence indicates its potential involvement in the pathogenesis and prognosis of DFUs. This review comprehensively explores the diverse roles of vitamin D in DFUs, encompassing its molecular mechanisms such as immunomodulation, promotion of angiogenesis, neuroprotection, and induction of antimicrobial peptides, as well as the metabolic characteristics associated with various vitamin D forms and compromised vitamin D receptor (VDR) signaling pathways. Although robust observational studies have established an association between vitamin D deficiency and adverse outcomes in DFUs, the clinical validation of supplementation efficacy through randomized controlled trials (RCTs) remains constrained by limitations such as small sample sizes, heterogeneity in study protocols, and insufficient long-term follow-up. This highlights the critical need for large-scale, high-quality studies to ascertain optimal treatment regimens and to cater to individualized patient requirements, particularly for individuals with obesity or those with renal impairments. Innovative strategies, such as the topical administration of vitamin D through intelligent delivery systems leveraging advanced biomaterials like nanofibers and hydrogels, exhibit substantial preclinical potential in enhancing stability, achieving targeted controlled release, and augmenting local biological effects, including the induction of antimicrobial peptides. Nevertheless, significant challenges persist in conclusively establishing clinical efficacy, comprehensively elucidating the underlying mechanisms, ensuring the safe translation of novel delivery systems, and developing personalized therapeutic strategies. The future success of these interventions hinges on meticulous research and interdisciplinary collaboration to seamlessly integrate validated vitamin D-based interventions into a comprehensive multidisciplinary management framework for DFUs, thereby holding promise for improving the clinical outcomes of this debilitating condition.

## 1. Introduction

Diabetic foot ulcers (DFUs), as defined by the latest guidelines of the International Working Group on the Diabetic Foot (IWGDF), are characterized by skin breakdown extending to at least the dermis in individuals with diabetes, often associated with diabetic peripheral neuropathy (DPN) and/or peripheral artery disease (PAD) [1]. DFUs constitute one of the most severe complications of diabetes, affecting approximately 6.3% of adults globally who have the condition [2,3]. This complication not only causes significant morbidity, including a lifetime amputation risk of approximately 20%, but also imposes a considerable economic burden due to elevated healthcare costs [4]. It is imperative to acknowledge that the development of DFUs is generally a chronic process, often becoming clinically apparent several years after the initial diagnosis of diabetes mellitus. A clinical study has demonstrated that a diabetes duration exceeding 10 years is associated with a 3.16-fold increased risk of developing foot ulcers [5]. Consequently, from a theoretical standpoint, diabetic patients have an adequate timeframe to implement preventive strategies aimed at avoiding the onset of foot ulcers. The underlying pathophysiology involves complex interactions between DPN, which causes sensory loss, foot deformities, and impaired skin function, and PAD, which contributes to tissue ischemia [4]. Collectively, these factors create a deleterious wound microenvironment characterized by impaired tissue regeneration, chronic inflammation, heightened susceptibility to infection, and prolonged ulceration [4,6]. Despite advancements in multidisciplinary treatment approaches, including debridement, offloading, revascularization, and infection management, the healing rates for DFUs remain suboptimal, with the recurrence rates reaching approximately 42% within one year and 65% within five years [7,8]. These findings underscore the critical need for innovative therapeutic strategies that specifically target the refractory mechanisms underlying DFU development and persistence.

In this context, vitamin D has garnered significant attention as an endogenous hormone with a wide array of biological functions. While traditionally recognized for its critical role in calcium phosphate metabolism and bone health [9], recent research underscores its substantial non-skeletal functions, which may be directly pertinent to key pathological aspects of DFUs [10]. Specifically, vitamin D exhibits the capacity to modulate immune responses, such as influencing macrophage polarization [11,12]; promoting angiogenesis potentially through the HIF-1α/VEGF pathway [12]; and inducing antimicrobial peptides, including LL-37 [13]. These functions present promising therapeutic opportunities for addressing the characteristic inflammatory imbalance, ischemia, and heightened susceptibility to infection observed in DFUs. Consistent clinical findings have demonstrated significantly lower levels of vitamin D in patients with DFUs [14,15], with some studies suggesting that vitamin D deficiency may serve as an independent risk factor for DFUs [14,16]. Although the precise causal relationship and underlying mechanisms warrant further investigation, preliminary evidence indicates that both systemic supplementation and the localized application of vitamin D hold potential therapeutic benefits for enhancing diabetic wound healing [12,17,18]. Nevertheless, it is crucial to emphasize that the development of DFUs is not a spontaneous process and vitamin D should not be considered as the sole determinant influencing the pathophysiology of DFUs. Behavioral and lifestyle factors, such as non-adherence to pharmacological diabetes treatment, suboptimal dietary practices, insufficient physical activity, and inadequate self-monitoring of blood glucose levels, often synergistically interact with genetic predispositions to modulate the pathological mechanisms underlying DFUs [7]. These factors may compromise tissue integrity and impair wound healing capabilities. However, given the potential multidimensional role of vitamin D in DFUs and the growing research interest, a comprehensive synthesis and critical evaluation of the current evidence are warranted. This review aims to systematically investigate the role of vitamin D in both the pathogenesis and therapeutics of DFUs. We investigate its underlying molecular mechanisms, critically evaluate the relevant clinical evidence, and explore innovative applications, with a particular focus on advanced biomaterial-based delivery systems. Ultimately, this work seeks to provide a robust scientific rationale and novel insights for the development of precise and effective intervention strategies for this challenging complication.

## 2. The Metabolic Profile of Vitamin D and DFUs

### 2.1. Different Forms of Vitamin D and Biological Significance

**Figure 1:**
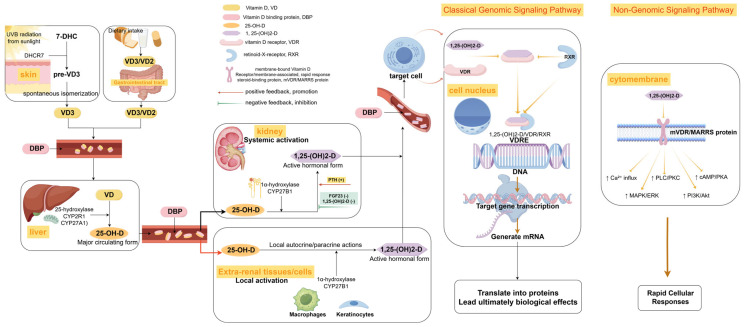
Schematic diagram of vitamin D metabolic activation and signaling pathways (by Figdraw). Vitamin D can be sourced from the endogenous synthesis of vitamin D3 (D3) in the skin via UVB irradiation, as well as from the dietary intake of vitamin D2 (D2) or D3. Upon entering the circulation, both D2 and D3 are transported by vitamin D binding protein (DBP) to the liver. There, enzymes such as CYP2R1 and CYP27A1 catalyze the formation of 25-hydroxyvitamin D [25(OH)D], which serves as the primary circulating form and an indicator of vitamin D status. Subsequently, in the kidneys, 25(OH)D is converted by CYP27B1 (1α-hydroxylase) into the active hormone 1,25(OH)2D. This renal conversion is stimulated by parathyroid hormone (PTH) and inhibited by fibroblast growth factor 23 (FGF23), as well as by 1,25(OH)2D itself through negative feedback mechanisms. Notably, various extrarenal tissues, including skin keratinocytes and macrophages, also express CYP27B1, facilitating the local synthesis of 1,25(OH)2D for autocrine and paracrine actions. CYP24A1 (24-hydroxylase) metabolizes both 25(OH)D and 1,25(OH)2D into inactive metabolites. The 1,25(OH)2D exerts its biological effects through both genomic and non-genomic mechanisms. In the non-genomic pathway, as a ligand, 1,25(OH)2D binds to the intracellular vitamin D receptor (VDR). The activated VDR forms a heterodimer with the Retinoid X Receptor (RXR), which subsequently binds to vitamin D response elements (VDREs) located in the promoter and enhancer regions of target genes. Through the recruitment of co-factors, including co-activators and co-repressors, the VDR/RXR complex modulates the transcription of target genes, thereby eliciting diverse biological effects pertinent to the pathophysiology of diabetic foot ulcers (DFUs), such as inducing antimicrobial peptides (AMPs), modulating immune responses, and influencing cell proliferation and differentiation. The non-genomic pathway involves the binding of 1,25(OH)2D to membrane-bound VDR (mVDR) or other membrane receptors (e.g., MARRS) on the cell surface, rapidly activating intracellular signaling cascades (e.g., Ca^2+^ influx, cAMP/PKA, PLC/PKC, MAPK/ERK, PI3K/Akt pathways), leading to rapid cellular responses.

After skin photoconversion or dietary intake, vitamin D undergoes sequential enzymatic hydroxylation [19,20]: It is first metabolized in the liver by CYP2R1 into 25-hydroxyvitamin D [25(OH)D], which represents the predominant circulating form of vitamin D. Subsequently, renal CYP27B1 catalyzes the synthesis of bioactive 1,25-dihydroxyvitamin D_3_ [1,25(OH)_2_D_3_]. The circulating 25(OH)D, characterized by its prolonged half-life of 2–3 weeks and stable concentration levels, serves as the gold-standard biomarker for assessing overall vitamin D status [21]. In contrast, 1,25(OH)_2_D_3_ exhibits rapid turnover kinetics, with a half-life of approximately 4 to 6 h, and it is tightly regulated by calcium-phosphorus homeostasis mechanisms, including parathyroid hormone (PTH) signaling [22,23]. This regulation ensures dynamic adaptation to physiological demands but complicates clinical interpretation due to transient fluctuations and sensitivity to feedback loops. Additionally, extrarenal tissues, such as macrophages and keratinocytes, autonomously express CYP27B1, enabling the localized production of 1,25(OH)_2_D_3_ through paracrine and autocrine pathways [24]. Notably, the skin plays a dual role in the photogenesis of vitamin D₃, involving the UVB-mediated conversion of 7-dehydrocholesterol and subsequent tissue-specific bioactivation. Cutaneous cells, such as keratinocytes, hair follicles, and Langerhans cells, independently hydroxylate 25(OH)D to 1,25(OH)_2_D_3_ without renal regulation [24]. This tissue-specific activation is essential for coordinating the reinforcement of the epithelial barrier, maintaining the balance between inflammation and repair, and regulating the expression of antimicrobial peptides [25,26,27]. Consequently, it represents a pivotal therapeutic target for remodeling the microenvironment of DFUs. This metabolic hierarchy and spatial heterogeneity highlight the distinct clinical significance of 25(OH)D, a stable biomarker, compared to 1,25(OH)_2_D_3_, a short-lived effector, in the pathophysiology of DFUs. This framework serves as the foundation for subsequent discussions on their concentration dissociation phenomena at systemic and tissue levels, along with local activation disorders. The metabolic pathway of vitamin D in the body is shown in Figure 1.

### 2.2. Characteristics of Vitamin D Metabolism in Patients with Diabetic Foot Ulcers

Large-scale clinical studies have consistently demonstrated significantly lower serum 25(OH)D levels in patients with DFUs [15]. A cross-sectional study [14] involving 1721 diabetic patients revealed that those with DFUs exhibited a median reduction of 21.3% in their 25(OH)D levels compared to non-DFU controls [35.80 nmol/L (IQR 26.19–48.09) vs. 45.48 nmol/L (IQR 33.44–59.25), *p* < 0.001]. Furthermore, patients with DFUs were found to have a 3.28-fold increased risk of severe vitamin D deficiency (<25 nmol/L; OR = 3.28, 95% CI 2.52–4.27) [15]. However, this association may be influenced by reverse causality, such as reduced sunlight exposure due to immobility, inadequate nutrient intake, and enhanced catabolism during infections, thus requiring prospective studies to clarify causal relationships. The bioactivity of vitamin D is determined by the bioavailable pool, which consists of free (approximately 0.03%) and albumin-bound (10–15%) fractions, while the vitamin D binding protein-bound (DBP-bound) fraction (85–90%) exhibits limited tissue uptake due to steric hindrance [28,29]. Consequently, conventional measurements of serum total 25(OH)D may overestimate the true biological activity of vitamin D. Additionally, polymorphisms in the vitamin D-binding protein (DBP), such as rs7041, rs4588, and rs2282679, influence the transport and bioavailability of vitamin D by inducing conformational changes that alter its binding affinity to DBP [30,31,32]. Studies have demonstrated that the polymorphisms rs7041 and rs4588 are associated with variations in DBP affinity [32]. Specific genotypes, such as Glu/Glu and Lys/Lys, are more prevalent among individuals with diabetes, potentially leading to reduced vitamin D levels and impaired insulin sensitivity [33,34], exacerbating the pathophysiological processes underlying DFUs. The aforementioned single nucleotide polymorphisms (SNPs) lead to amino acid substitutions within the DBP. This can alter the three-dimensional structure of the DBP binding pocket, thereby weakening its affinity for vitamin D. Consequently, this results in accelerated circulation clearance, decreased tissue delivery efficiency, and diminished bioavailability [30,32]. Additionally, certain genotypes, such as the CC genotype of rs7041, have been shown to correlate with elevated levels of pro-inflammatory cytokines [35], which may intensify the inflammatory microenvironment in DFUs. Furthermore, the rs2282679 locus is primarily associated with individual responsiveness to vitamin D supplementation [36].

### 2.3. Abnormal Vitamin D Receptor (VDR) Signaling

Beyond altered vitamin D metabolite levels, abnormalities in the VDR signaling pathway are increasingly recognized as critical aspects of the pathophysiology of DFUs. The expression and functionality of the VDR play a decisive role in cellular responsiveness to vitamin D. Notably, clinical studies have revealed significantly reduced VDR expression in wound margin tissues of patients with concurrent diabetic foot osteomyelitis (DFO) compared to those without it. Furthermore, both diminished serum 25(OH)D concentrations and reduced tissue VDR levels independently correlate with an increased risk of DFU/DFO and poorer healing rates [37]. Such downregulation of the VDR has also been observed in other diabetic complications, such as diabetic kidney disease (DKD), potentially attributable to multiple factors, including hyperglycemia, inflammation, and oxidative stress [38,39]. For instance, in vitro evidence indicates that high glucose conditions can suppress VDR expression via the upregulation of specific microRNAs, such as miR-125 b, thereby exacerbating neural cell injury [40]. Furthermore, polymorphisms in the VDR gene may influence VDR function and individual susceptibility to DFUs, although findings remain inconclusive. Systematic reviews and meta-analyses have suggested an association between the VDR rs2228570 polymorphism and an increased risk of DFUs [41,42]. One study associated the T allele of the FokI polymorphism with heightened levels of oxidative stress in patients with DFUs [43]. Additionally, a subsequent study established a significant association between the ApaI polymorphism in the VDR gene and DFUs, indicating that specific ApaI and BsmI genotypes are related to elevated levels of oxidative stress markers [44]. Conversely, conflicting evidence from other studies, including a case control study conducted in a South Indian population, failed to demonstrate any substantial associations between FokI, TaqI, or ApaI polymorphisms and either the risk of DFUs or vitamin D concentrations [45]. These inconsistencies likely arise due to variations in population characteristics and methodological differences in study design. Functionally, impaired VDR signaling leads to a direct reduction in the multifaceted protective effects of vitamin D. As a pivotal regulator in the induction of antimicrobial peptides such as LL-37/CAMP, VDR dysfunction compromises the innate immune response during wound healing [46,47]. Simultaneously, the VDR pathway plays an indispensable role in modulating inflammation, cell proliferation and differentiation, and autophagy processes, all of which are critical for the effective resolution of DFUs [38,39].

In summary, the metabolic profile of vitamin D in the context of DFUs is highly com-plex, characterized by the distinct biological roles of its various metabolites. Notably, 25(OH)D serves as a critical biomarker for assessing vitamin D status, whereas 1,25(OH)_2_D_3_ acts as the primary active mediator, including crucial local bioactivation within tissues such as the skin. Patients with DFUs consistently exhibit lower systemic levels of 25(OH)D, a phenomenon potentially influenced by genetic polymorphisms in the DBP, which can alter the bioavailability and transport of vitamin D. This alteration con-tributes to both systemic insufficiency and localized tissue dysfunction. Additionally, abnormalities in VDR signaling, including reduced VDR expression in the tissues of DFUs and the controversial effects of VDR gene polymorphisms, likely impair cellular responsiveness to vitamin D. This impairment exacerbates pathological processes and hinders effective wound healing.

## 3. Molecular Mechanism of Interaction Between Vitamin D and DFUs

### 3.1. Regulation of the Immune Microenvironment

Chronic inflammation in DFUs is intricately linked to an imbalance in macrophage polarization, specifically manifested by an elevated pro-inflammatory M1 phenotype and a reduced anti-inflammatory M2 phenotype [48]. Vitamin D plays a pivotal role in restoring this balance through multidimensional mechanisms. In hyperglycemic microenvironments, vitamin D inhibits M1 polarization by suppressing STAT-1 phosphorylation and TREM-1 expression [49]. Concurrently, it activates the VDR-PPARγ axis, thereby reversing the polarization of pro-inflammatory M1 macrophages and significantly diminishing the secretion of pro-inflammatory cytokines such as TNF-α, IL-6, and IL-1β [50]. Beyond its inhibitory effects on M1 macrophages, vitamin D promotes the transition from the M1 to the M2 phenotype through diverse pathways. For instance, in murine models, the topical application of vitamin D attenuates TLR4/NF-κB signaling, thus facilitating the shift of macrophages from the M1 to the M2 phenotype [51]. Additionally, vitamin D analogues enhance M2 conversion through the VDR/CYP2J2 axis [52]. Furthermore, vitamin D exhibits anti-inflammatory properties by modulating T-cell differentiation, which involves decreasing the proportion of Th1 cells and reducing IL-17 production [53].

Clinical studies have consistently demonstrated that patients with DFUs exhibit a significantly diminished expression of the antimicrobial peptide cathelicidin at wound margins [54]. Vitamin D insufficiency results in decreased levels of cathelicidin, thereby compromising local antimicrobial defenses and increasing vulnerability to infections [55]. This deficiency is further associated with an elevated risk of developing DFO [37]. Conversely, vitamin D supplementation may play a critical role in the management of diabetic foot infections by modulating the expression of antimicrobial peptides, such as cathelicidin (particularly its active form, LL-37) [56]. The active form of vitamin D, 1,25(OH)_2_D, binds to the VDR, inducing the synthesis of cathelicidin through the activation of CAMP gene transcription [46,57]. Clinical studies have established a correlation between vitamin D deficiency and an increased susceptibility to diabetic foot infections, as well as elevated levels of inflammatory cytokines [58,59,60]. Furthermore, vitamin D supplementation has been shown to enhance wound healing efficiency by 42% in patients with DFUs, while also improving markers of inflammation and oxidative stress, including hypersensitive C-reactive protein (CRP), the erythrocyte sedimentation rate, nitric oxide, and malondialdehyde [18]. In vitro mechanistic studies have revealed that active vitamin D not only promotes the synergistic expression of LL-37 and HBD-2, facilitating keratinocyte migration [46], but also mitigates excessive inflammation by upregulating IκBα (an inhibitor of NF-κB signaling) [61], thereby reducing the likelihood of secondary infections.

### 3.2. Angiogenesis and Ischemic Repair

Impaired angiogenesis and ischemic repair pose substantial challenges in the management of DFUs [62]. Vitamin D demonstrates significant pro-repair potential by targeting the hypoxia-inducible factor-1α (HIF-1α)/vascular endothelial growth factor (VEGF) pathway and restoring the functionality of endothelial progenitor cells (EPCs). In the context of DFUs, diabetes-induced inflammation and oxidative stress destabilize HIF-1α, thereby impeding adaptive angiogenesis [63]. The well-documented anti-inflammatory and antioxidant properties of vitamin D stabilize the HIF-1α protein, preserving its transcriptional activity [64,65]. This stabilization directly enhances the expression of VEGF and VEGFR2 [65,66], thus accelerating neovascularization—a phenomenon validated in diabetic wound models [12]. Furthermore, vitamin D optimizes this pathway by improving endothelial cell function, potentially through the modulation of endothelial nitric oxide synthase (eNOS) activity and the inhibition of excessive vascular cell adhesion molecule-1 (VCAM-1) expression [12,67]. Specifically, it regulates nitric oxide synthase (NOS) activity by enhancing eNOS function to ensure optimal NO bioavailability while simultaneously inhibiting iNOS overexpression to minimize the production of harmful NO derivatives [64,68]. Additionally, it activates the nuclear factor erythroid 2-related factor 2 (Nrf-2) signaling pathway, thereby promoting Nrf-2 nuclear translocation and upregulating downstream antioxidant genes [69,70].

Concurrently, vitamin D plays a critical role in rescuing compromised circulating EPCs, which are essential mediators of endogenous vascular repair. The diabetic microenvironment, particularly hyperglycemia and advanced glycation end products (AGEs), impairs EPC proliferation and migration while accelerating their senescence [71,72]. By interacting with its receptor, the VDR, vitamin D activates essential pro-survival and pro-migratory signaling pathways, such as the PI3K/AKT and ERK pathways, thereby effectively mitigating impairments induced by diabetes [73]. It has also been shown to significantly enhance EPC proliferation and tube formation in vitro, while potentially promoting their directed homing to ischemic sites, possibly through the CXCR4/SDF-1α chemokine axis [74,75]. Furthermore, vitamin D counteracts EPC oxidative stress and premature senescence by suppressing pro-inflammatory signals, including NF-κB, and by upregulating crucial antioxidant enzymes, such as superoxide dismutase (SOD) [64]. Emerging evidence further suggests that vitamin D has more profound regulatory effects, potentially involving epigenetic modifications, such as the modulation of specific miRNA expression [76], as well as enhancing paracrine signaling, including the stimulation of pro-angiogenic factor release [73].

### 3.3. Neuroprotective Effect

DPN represents one of the most critical factors contributing to the development of DFUs. It is imperative to acknowledge that DPN frequently predisposes individuals to neuropathic pain, a complex sensory disorder often characterized by hyperalgesia and allodynia [77]. Given the intricate pathophysiology of neuropathic pain, which encompasses a diverse array of neurotransmitter systems such as serotonergic, glutamatergic, GABAergic, and glycinergic pathways, as well as various ion channels [78], this discussion will focus on aspects pertinent to the interactions with vitamin D. In diabetic rat models, the vitamin D3 derivative CB1093 has been demonstrated to upregulate nerve growth factor (NGF) gene expression, thereby preventing the depletion of neuronal target gene products [79]. Furthermore, in diabetic mouse models, the topical application of active vitamin D has been shown to enhance the protein levels of NGF, brain-derived neurotrophic factor (BDNF), and neurotrophin-3 (NT-3), which accelerates nerve regeneration [80]. Additionally, vitamin D has protective effects on peripheral nerve fibers by alleviating demyelination and promoting axon regeneration [81]. The overactivation of specific channels, such as TRPV1, plays a pivotal role in the development of hyperalgesia, which constitutes a key component of neuropathic pain associated with DPN. Vitamin D may downregulate TRPV1 activity by inhibiting the production of cytokines, such as TNF-α and INFγ, through mechanisms independent of the VDR [82]. Animal studies have further confirmed that vitamin D analogues, such as EB1089, can effectively increase pain thresholds by modulating TRPV1 phosphorylation [83,84]. Moreover, vitamin D plays a critical role in neuronal protection by stabilizing mitochondrial membrane potential and exerting anti-inflammatory and antioxidative stress effects, thereby reducing neuronal apoptosis and damage [85,86]. These mechanistic insights derived from basic research are corroborated by clinical evidence demonstrating that vitamin D deficiency constitutes an independent risk factor for the development of DPN [87], Notably, its levels exhibit a significant correlation with the severity of DPN and reduced nerve conduction velocity, as evidenced by studies involving a cohort of 1192 patients [88]. Additionally, patients suffering from painful DPN generally present with significantly lower vitamin D levels than those without pain [89], which aligns with the established role of vitamin D in modulating the TRPV1 pathway. Importantly, clinical intervention studies have revealed that vitamin D supplementation not only enhances nerve conduction velocity but also alleviates pain symptoms and improves neuropathy-related quality of life (NeuroQoL) in patients with DPN [90].

**Figure 2:**
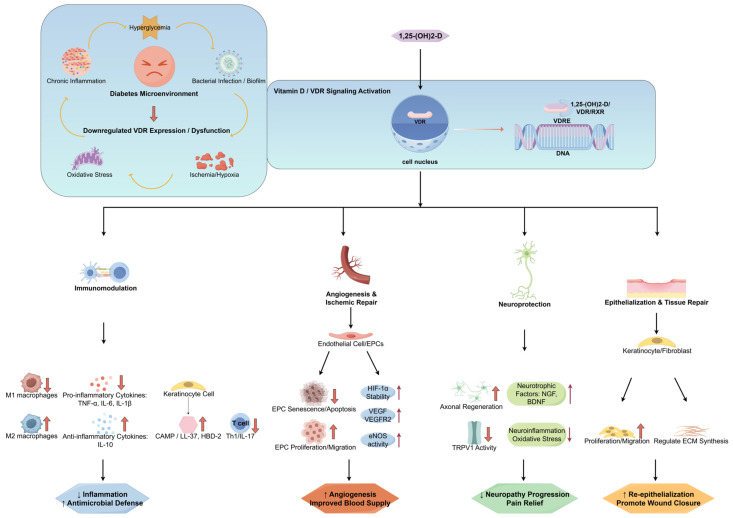
Mechanistic network of the pleiotropic regulatory effects of the vitamin D/VDR signaling pathway in the pathophysiological microenvironment of DFUs (by Figdraw). The complex microenvironment of diabetic foot ulcers (DFUs), characterized by hyperglycemia, ischemia/hypoxia, chronic inflammation, oxidative stress, and bacterial infection, frequently results in the downregulated expression or dysfunction of the vitamin D receptor (VDR). This impairment undermines the physiological effects of vitamin D. Activation of the vitamin D/VDR signaling pathway may exert beneficial influences on DFUs through the following mechanisms: (1) Immunomodulation: It inhibits macrophage polarization towards the pro-inflammatory M1 phenotype while promoting the anti-inflammatory M2 phenotype. Consequently, this reduces the secretion of pro-inflammatory cytokines (e.g., TNF-α and IL-6) and enhances the expression of antimicrobial peptides (AMPs, e.g., CAMP/LL-37) by keratinocytes and other cell types, thereby attenuating inflammation and strengthening antimicrobial defense. (2) Angiogenesis and Ischemic Repair: Activated vitamin D promotes neovascularization and improves tissue perfusion by stabilizing hypoxia-inducible factor-1α (HIF-1α), upregulating vascular endothelial growth factor (VEGF) and its receptor (VEGFR2), and enhancing the proliferation, migration, and survival of endothelial progenitor cells (EPCs). (3) Neuroprotection: Activated vitamin D increases the expression of neurotrophic factors (e.g., NGF and BDNF), promotes axonal regeneration, and potentially alleviates neuropathy progression and pain by modulating transient receptor potential vanilloid 1 (TRPV1) activity while suppressing neuroinflammation and oxidative stress.

The effects of the vitamin D/VDR signaling pathway in the pathophysiological microenvironment of DFUs are shown in Figure 2.

In summary, vitamin D exerts multifaceted beneficial effects on the microenvironment of DFUs through interconnected molecular mechanisms. It rebalances immune responses by facilitating the polarization shift from pro-inflammatory M1 to anti-inflammatory M2 macrophages and enhancing the production of antimicrobial peptides, thereby effectively combating infection and chronic inflammation. Concurrently, vitamin D promotes angiogenesis and ischemic repair through the stabilization of HIF-1α, VEGF, and the restoration of EPC function. Furthermore, its neuroprotective actions, including the upregulation of neurotrophic factors and modulation of nociceptive pathways such as TRPV1, address the critical DPN component associated with DFUs. These integrated actions underscore the potential of vitamin D as a pleiotropic agent for improving DFU healing by targeting key pathological processes.

## 4. Application of Vitamin D in the Clinical Management of DFUs

### 4.1. Association Studies on Vitamin D Status with DFU Risk and Prognosis

Accumulating clinical evidence highlights a significant association between vitamin D deficiency and the risk, severity, and prognosis of DFUs. Although individual cross-sectional studies on vitamin D status in DFUs exhibit heterogeneity, pooled analyses, particularly a recent meta-analysis encompassing 36 studies with 11,298 individuals, consistently indicate significantly lower serum 25(OH)D levels in patients with DFUs (with a mean difference of approximately −10.93 nmol/L) and a substantially elevated risk of DFUs for patients with vitamin D deficiency/severe deficiency (with an OR range of approximately 2.25–3.28) [15]. Furthermore, VDR gene polymorphisms may contribute to individual susceptibility. A case control study conducted in Southern India found that VDR gene polymorphisms, such as Fokl, Taql, and Apal, might influence individual susceptibility to DFUs, suggesting a genetic modulation of vitamin D metabolism [45]. Prospective cohort studies have provided additional evidence regarding the impact of vitamin D status on DFU progression and outcomes. Vitamin D levels are positively correlated with DFU healing rates and inversely associated with the likelihood of deep tissue infection, osteomyelitis, or progression to higher-grade ulcers [14,91]. Crucially, vitamin D status significantly influences the ultimate outcomes of DFUs. Patients with lower baseline serum 25(OH)D levels tend to have poorer prognoses during follow-up, including delayed wound healing [91]. Lower levels are not only associated with slower granulation tissue formation and prolonged healing times but are also linked to adverse outcomes such as recurrence and amputation [37]. A prospective cohort study involving 275 patients demonstrated that each 1 nmol/L decrease in the serum vitamin D concentration was associated with a 2.1% increased risk of all-cause mortality, potentially attributable to chronic inflammation and cardiovascular risk [92].

### 4.2. Effect of Systemic Vitamin D Supplementation on DFUs

Interventional studies, particularly randomized controlled trials (RCTs) and their meta-analyses [18,93], have provided preliminary evidence supporting the potential therapeutic benefits of systemic vitamin D supplementation for DFUs. These benefits are likely mediated through the pleiotropic mechanisms previously outlined, such as immune modulation, angiogenesis promotion, and neuroprotection. Notably, published RCTs predominantly report favorable outcomes following vitamin D supplementation. For instance, studies employing high-dose intermittent oral regimens (e.g., 50,000 IU bi-weekly or 60,000 IU weekly for 12 weeks) compared to placebo have demonstrated improvements in inflammatory and metabolic markers, along with significant reductions in ulcer area and other healing parameters [94,95]. Further, additional research has indicated that single high-dose intramuscular injections (300,000 IU) or long-term, higher-dose daily oral intake (e.g., 6800 IU/day for 48 weeks) may be more effective than lower doses or placebo in enhancing vitamin D status and accelerating ulcer healing rates [96,97]. However, although these findings are promising, their reliability and generalizability remain significantly limited. This limitation is primarily attributed to small sample sizes; potential methodological flaws, including variable study quality across trials; and substantial heterogeneity in vitamin D dosage regimens and treatment durations. Such heterogeneity impedes a robust quantification of efficacy and complicates the establishment of a standardized, evidence-based optimal supplementation protocol. Moreover, vitamin D supplementation strategies should be individually tailored to specific populations. For instance, individuals with obesity, who experience the sequestration of vitamin D in adipose tissue, often require higher doses of conventional vitamin D to achieve target serum levels [98]. In contrast, patients with moderate-to-severe chronic kidney disease (CKD), due to impaired renal activation of vitamin D, typically require direct supplementation with active vitamin D analogues, such as calcitriol [99]. This therapeutic approach necessitates the close monitoring of calcium, phosphate, and PTH levels to minimize potential risks [100].

### 4.3. Innovative Exploration of Topical Application of Vitamin D or Its Analogues

Given the uncertainties regarding dose–efficacy relationships, significant inter-individual variability, and potential safety considerations, particularly in specific populations, associated with systemic vitamin D supplementation for DFUs, the topical application of vitamin D or its active analogues represents an emerging and promising area for innovative investigation. This strategy seeks to achieve higher local concentrations of vitamin D while minimizing systemic exposure by directly targeting the wound microenvironment, thereby potentially enhancing its pro-healing properties with greater precision. The active form of vitamin D and its synthetic analogues, such as calcipotriol, are recognized for their ability to directly activate the VDR in skin cells, including keratinocytes, fibroblasts, and immune cells [25,101]. Their capacity to modulate cell proliferation and differentiation, suppress local inflammation, and regulate immune responses has been firmly established through extensive application in topical treatments for dermatological conditions such as psoriasis [102]. By leveraging the well-documented mechanisms of active vitamin D and its analogues on skin cells, this innovative approach to treating diabetic wounds focuses on direct effects within the wound area, similarly to systemic supplementation. Although high-level clinical evidence for topical vitamin D in the treatment of DFUs continues to accumulate, preliminary innovative explorations, including animal model experiments and in vitro cell studies, have begun to demonstrate promising results [51,80,103]. Although clinical observations suggest reduced VDR expression in the marginal tissues of DFUs, the topical application of high-concentration vitamin D may still represent a potentially rational therapeutic strategy. This approach could exert its effects through potential upregulation of VDR, activation of non-genomic pathways, and effective engagement of residual VDR to initiate positive feedback repair mechanisms, as outlined earlier in this article. These studies have not only explored the direct application of topically active vitamin D but have also laid the foundation for advanced delivery strategies, such as functionalized dressings, in subsequent phases. Current research prioritizes validating the efficacy and safety of these topical approaches while determining the optimal drug form (prodrug versus active analogue), concentration, formulation, and treatment regimen.

In summary, the clinical application of vitamin D in the management of DFUs is multifaceted. Observational studies consistently demonstrate an association between vitamin D deficiency and an increased risk, severity, and adverse outcomes of DFUs, underscoring the critical importance of monitoring vitamin D status. Although systemic vitamin D supplementation has shown positive effects in some RCTs, the optimal regimen remains undefined due to considerable heterogeneity in study designs and the necessity for individualized approaches, particularly in obese or renally impaired patients. The innovative topical application of vitamin D or its analogues is emerging as a promising strategy to enhance local efficacy while minimizing systemic risks. However, there is an urgent need for large-scale, rigorously designed RCTs to conclusively establish efficacy, determine optimal dosages, and define supplementation schedules. Future studies should prioritize these recommendations.

## 5. Application of Vitamin D-Functionalized Materials in Wound Healing

The inherent challenges of delivering vitamin D through traditional systemic administration and simple topical application encompass suboptimal bioavailability, insufficient targeting precision, compromised stability, and unregulated release kinetics. To fully exploit the localized therapeutic potential of vitamin D, the development of intelligent delivery systems based on advanced biomaterials has become a dynamic and promising area in the therapeutic research of DFUs. Through sophisticated material engineering, these systems aim to enhance drug delivery efficiency; address stability issues for vitamin D, particularly active forms such as 1,25(OH)2D3; and enable controlled, sustained release within the wound microenvironment to amplify biological efficacy.

Electrospun nanofibers have emerged as a principal modality under investigation. For instance, polycaprolactone (PCL) nanofibers loaded with active vitamin D (1,25(OH)_2_D_3_) exhibit high encapsulation efficiency and facilitate sustained release over several weeks [104]. Notably, this system consistently induces a robust expression of the endogenous antimicrobial peptide hCAP18/LL-37 (CAMP) across diverse models, such as in vitro cultures, in vivo humanized mouse wounds, and ex vivo human skin, providing a material-driven strategy to bolster innate immune defense against wound infection [47].

**Figure 3:**
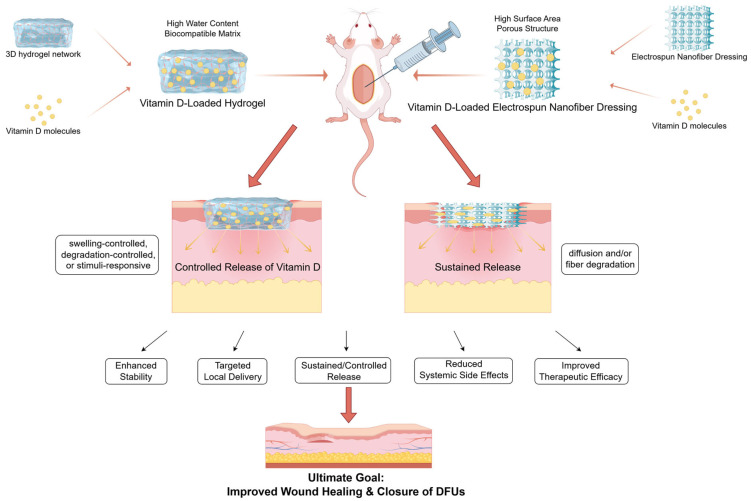
Schematic illustration of the locally delivered vitamin D strategies utilizing advanced biomaterials (by Figdraw).

Complementing nanofibrous approaches, hydrogels serve as an additional critical modality for the delivery of vitamin D. Hydrogels derived from natural polymers, such as alginate, hyaluronic acid, and even rice flour, effectively encapsulate vitamin D, enhance its stability, and function as reservoirs for sustained release [105,106,107]. The therapeutic potential of these hydrogels has been preliminarily demonstrated in animal models; specifically, alginate hydrogels loaded with a defined dose of vitamin D3 significantly accelerated the healing of full-thickness skin defects by promoting wound closure, re-epithelialization, and granulation tissue formation [105]. In vitro studies further suggest that hyaluronic acid-based hydrogels delivering vitamin D may mitigate inflammatory damage [106]. Furthermore, the incorporation of polymer nanoparticles, such as PLGA carrying 1,25(OH)_2_D_3_, into hydrogel matrices generates more sophisticated composite systems. Figure 3 presents a schematic illustration of the locally delivered vitamin D strategy utilizing advanced biomaterials. These systems have exhibited anti-inflammatory and anti-proliferative effects in vascular restenosis models, offering conceptual insights for the treatment of DFUs [108,109]. Although stimuli-responsive hydrogels theoretically enable smarter, on-demand release mechanisms, their specific application for vitamin D delivery is still in its nascent stages.

Although preclinical data for nanofiber-based and hydrogel-based vitamin D intelligent delivery systems are promising, their clinical translation necessitates careful evaluation. The heterogeneity of DFUs, characterized by varying severity, infection, ischemia, and biofilm formation, requires delivery platforms with tailored release kinetics and bioactivities. Consequently, future material engineering should increasingly focus on customization or responsive modulation according to the specific pathophysiological state of the wound to achieve more precise and individualized therapies. Furthermore, thorough validation of long-term biocompatibility and safety within the diabetic environment, scalable and cost-effective manufacturing processes, as well as patient-related factors, including treatment acceptance and clinical compliance, are critical for successful clinical adoption.

In summary, the current research on intelligent delivery systems that leverage advanced materials such as nanofibers and hydrogels clearly demonstrates substantial potential for effectively modulating critical biological processes associated with wound healing, including innate immunity and tissue repair. This is achieved by enhancing the stability and release kinetics of vitamin D. These technological platforms provide a vital foundation for the development of more efficacious localized therapies for DFUs. However, the successful translation of these innovations into widely adopted clinical therapies depends not only on continued technological advancements but also on a profound understanding of wound complexity and the resolution of numerous practical translational challenges, ultimately confirmed through rigorous clinical trials.

## 6. Challenges and Future Research Directions

While the role of vitamin D in the pathophysiology and potential treatment of DFUs is increasingly acknowledged, translating current knowledge into reliable and effective clinical strategies encounters several critical scientific and practical challenges, thereby defining the core agenda for future research.

First, at the fundamental research level, a deeper and more comprehensive understanding of the precise mechanisms of action of vitamin D within the complex microenvironment of DFUs is critical. Although its pleiotropic functions, including immunomodulation, pro-angiogenesis, and neuroprotection, have been well established, significant gaps in knowledge remain regarding its intricate roles in the pathogenesis and healing processes of DFUs. Specifically, further research is required to elucidate its cell-type-specific effects on various immune subsets and senescent cells; assess the relative significance of different signaling pathways; and characterize its complex interactions with other molecules, such as growth factors and cytokines, in this context. Achieving this necessitates the integration of advanced technologies, such as omics approaches, high-resolution imaging techniques, sophisticated biomimetic models like organ-on-a-chip platforms, and refined animal systems. Elucidating these detailed mechanisms is fundamental for the development of more targeted and effective therapeutic strategies.

Second, to more comprehensively contextualize the current scientific landscape and pinpoint specific unmet needs, we performed an in-depth comparative analysis between this review and selected previous literature on vitamin D and DFUs, as presented in Table 1. This analysis highlights the varying primary emphases, the text of mechanistic discussions, and the dynamic progression of research within this domain, thereby underscoring the persistent challenges associated with clinical translation. Chief among these is the paucity of high-quality interventional evidence, which represents a significant bottleneck. There is an urgent requirement for large-scale, multicenter, rigorously designed RCTs to definitively evaluate the net benefits, safety profiles, and optimal dosing regimens of vitamin D supplementation, whether administered systemically or topically. Furthermore, the successful clinical translation of advanced material-based local vitamin D delivery systems encounters significant translational barriers. Comprehensive preclinical studies are crucial to thoroughly assess their long-term in vivo biocompatibility, safety, predictable drug release kinetics, and robust efficacy within the complex wound microenvironment. Additionally, the development of personalized therapeutic strategies constitutes a critical future direction for enhancing treatment efficacy. Considering the considerable inter-patient heterogeneity, such as genetic background, comorbidities, and baseline vitamin D status, future research should prioritize the identification of predictive biomarkers and the establishment of individualized risk stratification and treatment paradigms.

Third, interdisciplinary collaboration is indispensable for addressing these challenges and advancing the field. Future advancements will depend heavily on close cooperation and knowledge exchange between basic scientists, materials engineers, and clinicians. Notably, the insights derived from vitamin D-related research and potential therapeutic strategies must be seamlessly integrated into the existing multidisciplinary team (MDT) framework for DFU care, which encompasses relevant departments, including endocrinology, orthopedics, burn surgery, vascular surgery, ultrasound diagnostics, and imaging services. This will ensure synergy with conventional treatments such as debridement, offloading, infection control, and revascularization. Furthermore, interdisciplinary innovations, including the application of artificial intelligence for advanced data analysis and the development of innovative biomaterials, will play a pivotal role in driving progress.

Looking ahead, to effectively address these challenges and fully harness the therapeutic potential of vitamin D, a profound integration and synergistic innovation across basic science, materials engineering, and clinical medicine are indispensable. By means of rigorous clinical validation, in-depth mechanistic investigation, advancement in delivery technologies, and strategic incorporation within the multidisciplinary team (MDT) framework, vitamin D holds promise as a valuable and cost-effective component in the comprehensive management strategy for DFUs, thereby ultimately enhancing patient outcomes.

## 7. Conclusions

Vitamin D, through its pleiotropic biological functions encompassing immunomodulation, pro-angiogenesis, and neuroprotection, contributes to the complex pathophysiology of DFUs. Robust observational evidence consistently demonstrates an association between vitamin D deficiency and both an elevated risk and poorer prognosis of DFUs. While both systemic supplementation and innovative topical/biomaterial-based delivery of vitamin D exhibit therapeutic promise in preclinical studies and some clinical settings, their definitive clinical efficacy and optimal application necessitate validation through large-scale, rigorously designed RCTs. Future research should focus on deepening the mechanistic understanding of vitamin D’s actions within the DFU microenvironment and developing strategies for personalized interventions. This must account for the heterogeneity of patient responses and the multifactorial nature of DFU pathogenesis. Crucially, while vitamin D modulation may serve as a valuable adjunctive approach, its potential impact must be contextualized within the broader spectrum of DFU management. Addressing hyperglycemia, neuropathy, and peripheral artery disease and ensuring adherence to comprehensive care remain paramount. A critical future direction involves investigating how best to integrate targeted vitamin D strategies into existing multidisciplinary preventative and treatment frameworks for DFUs, rather than considering it as a standalone solution. Furthermore, given the devastating consequences of DFUs, future efforts must emphasize not only treatment but also the potential role of maintaining vitamin D sufficiency as a part of preventative strategies within a multidisciplinary DFU management framework, with prevention prioritized over cure. Overcoming current evidentiary and translational challenges through sustained rigorous research and technological innovation is essential to fully elucidate and leverage the true clinical benefits of vitamin D for patients at risk of or suffering from this debilitating complication.

## Figures and Tables

**Table 1:** Comparative Analysis of the Current Review with Selected Previous Literature on Vitamin D and Diabetic Foot Ulcers.

Comparison Aspect	Tang et al., 2025 (Current Review)	Macido, 2018 [110]	Greenhagen et al., 2019 [111]	Kurian et al., 2021 [112]	Putz et al., 2022 [90]	Liu et al., 2024 [113]
**Title**	The Multi-Dimensional Role of Vitamin D in the Pathophysiology and Treatment of Diabetic Foot Ulcers: from Molecular Mechanisms to Clinical Translation	Diabetic Foot Ulcers and Vitamin D Status: A Literature Review	Serum vitamin D and diabetic foot complications	Vitamin D Supplementation in Diabetic Foot Ulcers: A Current Perspective	Vitamin D in the Prevention and Treatment of Diabetic Neuropathy	The role of vitamin D in diabetic foot ulcer; an umbrella review of meta-analyses
**Article Type**	Narrative Review	Literature Review	Original Research	Narrative Review	Narrative Review	Umbrella Review of Meta-Analyses
**Primary Theme**	The multidimensional, integrated role and translation of vitamin D in DFUs: from molecular mechanisms to biomaterial applications	A early review on vitamin D status and DFUs/DFI links, highlighting insufficient evidence	Clinical association between vitamin D levels with diabetic foot complications in a specific cohort	The effects of vitamin D on DFUs risk factors and healing, as well as supplementation considerations	The specific link between vitamin D deficiency and diabetic neuropathy, as well as supplementation perspective	Summary and systematic quality assessment of the existing meta-analytic evidence on the association of vitamin D and DFUs
**Molecular Mechanisms**	Discusses in detail the specific roles of immune, vascular, neuro, AMPs in DFUs microenvironment	Summarizes the metabolic pathways of vitamin D and its effects on skin cells (keratinocytes and fibroblasts)	Limited	General overview of immune, vascular, neurological mechanisms	Focuses on the investigation of neuroprotective mechanisms	Mentions immunomodulation as background in discussion
**Vitamin D Forms and VDR**	Analyzes the different forms of vitamin D, and the dysregulation, polymorphisms and dysfunction of VDR	Mentions the physiological metabolism of vitamin D and VDR	Mentions only serum 25(OH)D	Mentions vitamin D metabolism and VDR, but no further discussion is made	Focuses on the VDR of nervous system rather than the systemic one	Mentions only serum 25(OH)D
**Biomaterials and Drug Delivery**	Detailed discussion of intelligent delivery systems of vitamin D, including nanofibers and hydrogels	Not covered	Not covered	Not covered	Not covered	Not covered
**Translational Medicine**	Emphasizes basic-clinical-materials translational pathway and MDT integration	Literature review and initial inference	Clinical observation research	Links physiological mechanisms with clinical supplementation	Links neuropathology with clinical supplementation	Systematic assessment of clinical evidence, guiding future translational research directions
**Consideration of Special Populations**	Explicitly discusses the individualized strategies for obese and renal insufficiency populations	Not specifically discussed	Not specifically discussed	Briefly mentions it in the conclusion	Mentions the lack of consideration of the differences among the population in the limitations of discussion	Not directly involved
**Main Findings and Conclusions**	Explores the multi-mechanistic impact of vitamin D on DFUs, notes supplementation potential despite lacking strong evidence, highlights material delivery as innovation, and calls for high-quality RCTs	Vitamin D deficiency may be associated with DFUs and foot infections, but evidence is minimal; more research is needed	Lower vitamin D levels are associated with PAD, DFI, and DFUs in diabetic patients; no significant difference for patients with CN	Vitamin D has protective roles in immune systems, vascular systems and wound healing; it could be a preferred adjuvant in the management of DFUs	Vitamin D deficiency may play roles in DPN, DFUs and CAN; supplementation is effective for neuropathic pain, may slow neural damage	Pooled meta-analyses suggest that low vitamin D levels are linked to DFU risk, and supplementation may be beneficial; the results need to be interpreted with caution due to the potential bias

Note: Abbreviations: DFUs: Diabetic Foot Ulcers; VDR: Vitamin D Receptor; PAD: Peripheral Artery Disease; DPN: Diabetic Peripheral Neuropathy; CN: Charcot Neuroarthropathy; DFI: Diabetic Foot Infection; AMPs: Antimicrobial Peptides; RCTs: Randomized Controlled Trials; MDT: Multidisciplinary Team.

## References

[1] van Netten J.J., Bus S.A., Apelqvist J., Chen P., Chuter V., Fitridge R., Game F., Hinchliffe R.J., Lazzarini P.A., Mills J. (2024). Definitions and criteria for diabetes-related foot disease (IWGDF 2023 update). Diabetes Metab. Res. Rev..

[2] Zhang P., Lu J., Jing Y., Tang S., Zhu D., Bi Y. (2016). Global epidemiology of diabetic foot ulceration: A systematic review and meta-analysis. Ann. Med..

[3] Zhang Y., Lazzarini P.A., McPhail S.M., van Netten J.J., Armstrong D.G., Pacella R.E. (2020). Global Disability Burdens of Diabetes-Related Lower-Extremity Complications in 1990 and 2016. Diabetes Care.

[4] McDermott K., Fang M., Boulton A.J.M., Selvin E., Hicks C.W. (2022). Etiology, Epidemiology, and Disparities in the Burden of Diabetic Foot Ulcers. Diabetes Care.

[5] Almobarak A.O., Awadalla H., Osman M., Ahmed M.H. (2017). Prevalence of diabetic foot ulceration and associated risk factors: An old and still major public health problem in Khartoum, Sudan?. Ann. Transl. Med..

[6] Fife C.E., Eckert K.A., Carter M.J. (2018). Publicly Reported Wound Healing Rates: The Fantasy and the Reality. Adv. Wound Care.

[7] Armstrong D.G., Boulton A.J.M., Bus S.A. (2017). Diabetic Foot Ulcers and Their Recurrence. N. Engl. J. Med..

[8] Armstrong D.G., Tan T.W., Boulton A.J.M., Bus S.A. (2023). Diabetic Foot Ulcers: A Review. JAMA.

[9] Kamiński A., Bogacz A., Niezgoda-Nowak J.T., Podralska M., Górska A., Soczawa M., Czerny B. (2025). The VDR rs1544410 and rs11568820 Variants and the Risk of Osteoporosis in the Polish Population. Int. J. Mol. Sci..

[10] Bouillon R., Marcocci C., Carmeliet G., Bikle D., White J.H., Dawson-Hughes B., Lips P., Munns C.F., Lazaretti-Castro M., Giustina A. (2019). Skeletal and Extraskeletal Actions of Vitamin D: Current Evidence and Outstanding Questions. Endocr. Rev..

[11] Zhu W., Zhu Y., Zhang S., Zhang W., Si Z., Bai Y., Wu Y., Fu Y., Zhang Y., Zhang L. (2023). 1,25-Dihydroxyvitamin D regulates macrophage activation through FBP1/PKR and ameliorates arthritis in TNF-transgenic mice. J. Steroid Biochem. Mol. Biol..

[12] Ma Y., Gong Y., Wu Y., Li Y., Fu R., Zhang X., Zhao Q., Zhi X. (2024). 1,25(OH)_2_D_3_ improves diabetic wound healing by modulating inflammation and promoting angiogenesis. J. Steroid Biochem. Mol. Biol..

[13] Vaccaro J.A., Qasem A., Naser S.A. (2022). Cathelicidin Mediates an Anti-Inflammatory Role of Active Vitamin D (Calcitriol) During M. paratuberculosis Infection. Front. Cell. Infect. Microbiol..

[14] Tang W., Chen L., Ma W., Chen D., Wang C., Gao Y., Ran X. (2022). Association between vitamin D status and diabetic foot in patients with type 2 diabetes mellitus. J. Diabetes Investig..

[15] Tang W., Chen D., Chen L., Liu G., Sun S., Wang C., Gao Y., Ran X. (2024). The correlation between serum vitamin D status and the occurrence of diabetic foot ulcers: A comprehensive systematic review and meta-analysis. Sci. Res..

[16] Atoum M.F., Al Shdaifat A., Al Hourani H., Al Hyari M., Zahran R., Abu Shaikh H. (2023). Relationship of Serum Vitamin D Levels With Diabetic Foot in Patients With Type 2 Diabetes Mellitus: A Cross-Sectional Study. Int. J. Low. Extremity Wounds.

[17] Tian X.Q., Chen T.C., Holick M.F. (1995). 1,25-dihydroxyvitamin D_3_: A novel agent for enhancing wound healing. J. Cell. Biochem..

[18] Wu X., Zeng J., Ye X., Peng M., Lan Y., Zhang S., Li H. (2025). Effects of vitamin D supplementation on diabetic foot ulcer healing: A meta-analysis. Postgrad. Med. J..

[19] Bouillon R., Manousaki D., Rosen C., Trajanoska K., Rivadeneira F., Richards J.B. (2022). The health effects of vitamin D supplementation: Evidence from human studies. Nat. Rev. Endocrinol..

[20] Delrue C., Speeckaert M.M. (2023). Vitamin D and Vitamin D-Binding Protein in Health and Disease. Int. J. Mol. Sci..

[21] Herrmann M., Zelzer S., Cavalier E., Kleber M., Drexler-Helmberg C., Schlenke P., Curcic P., Keppel M.H., Enko D., Scharnagl H. (2023). Functional Assessment of Vitamin D Status by a Novel Metabolic Approach: The Low Vitamin D Profile Concept. Clin. Chem..

[22] Jin Z., Bertholf R.L., Yi X. (2023). Advances and challenges in the measurement of 1,25-dihydroxyvitamin D: A comprehensive review. Crit. Rev. Clin. Lab. Sci..

[23] Yoon S.H., Meyer M.B., Arevalo C., Tekguc M., Zhang C., Wang J.S., Castro Andrade C.D., Strauss K., Sato T., Benkusky N.A. (2023). A parathyroid hormone/salt-inducible kinase signaling axis controls renal vitamin D activation and organismal calcium homeostasis. J. Clin. Investig..

[24] Fuchs M.A., Grabner A., Shi M., Murray S.L., Burke E.J., Latic N., Thiriveedi V., Roper J., Ide S., Abe K. (2024). Intestinal Cyp24a1 regulates vitamin D locally independent of systemic regulation by renal Cyp24a1 in mice. J. Clin. Investig..

[25] Reichrath J., Zouboulis C.C., Vogt T., Holick M.F. (2016). Targeting the vitamin D endocrine system (VDES) for the management of inflammatory and malignant skin diseases: An historical view and outlook. Rev. Endocr. Metab. Disord..

[26] Slominski A.T., Brożyna A.A., Zmijewski M.A., Jóźwicki W., Jetten A.M., Mason R.S., Tuckey R.C., Elmets C.A. (2017). Vitamin D signaling and melanoma: Role of vitamin D and its receptors in melanoma progression and management. Lab. Investig..

[27] Chaiprasongsuk A., Janjetovic Z., Kim T.K., Tuckey R.C., Li W., Raman C., Panich U., Slominski A.T. (2020). CYP_11_A_1_-derived vitamin D_3_ products protect against UVB-induced inflammation and promote keratinocytes differentiation. Free Radic. Biol. Med..

[28] Bikle D.D., Schwartz J. (2019). Vitamin D Binding Protein, Total and Free Vitamin D Levels in Different Physiological and Pathophysiological Conditions. Front. Endocrinol..

[29] Alexandridou A., Stokes C.S., Volmer D.A. (2025). Measurement of Serum Free Vitamin D Concentrations: Importance, Challenges, and the Emerging Role of Mass Spectrometry. Clin. Chem..

[30] Rozmus D., Ciesielska A., Płomiński J., Grzybowski R., Fiedorowicz E., Kordulewska N., Savelkoul H., Kostyra E., Cieślińska A. (2020). Vitamin D Binding Protein (VDBP) and Its Gene Polymorphisms-The Risk of Malignant Tumors and Other Diseases. Int. J. Mol. Sci..

[31] Karcioglu Batur L., Hekim N. (2021). The role of DBP gene polymorphisms in the prevalence of new coronavirus disease 2019 infection and mortality rate. J. Med. Virol..

[32] Rozmus D., Płomiński J., Augustyn K., Cieślińska A. (2022). rs7041 and rs4588 Polymorphisms in Vitamin D Binding Protein Gene (VDBP) and the Risk of Diseases. Int. J. Mol. Sci..

[33] Rahman M.M., Hosen M.B., Faruk M.O., Hasan M.M., Kabir Y., Howlader M.Z.H. (2017). Association of vitamin D and vitamin D binding protein (DBP) gene polymorphism with susceptibility of type 2 diabetes mellitus in Bangladesh. Gene.

[34] Setayesh L., Casazza K., Moradi N., Mehranfar S., Yarizadeh H., Amini A., Yekaninejad M.S., Mirzaei K. (2021). Association of vitamin D-binding protein and vitamin D_3_ with insulin and homeostatic model assessment (HOMA-IR) in overweight and obese females. BMC Res. Notes.

[35] Bastyte D., Tamasauskiene L., Stakaitiene I., Briede K., Ugenskiene R., Valiukeviciene S., Gradauskiene B. (2024). Relation of T Cell Profile with Vitamin D Receptor and Vitamin D-Binding Protein Gene Polymorphisms in Atopy. Int. J. Mol. Sci..

[36] Asghari G., Yuzbashian E., Nikparast A., Najd Hassan Bonab L., Mahdavi M., Daneshpour M.S., Hosseinpanah F., Mirmiran P. (2022). Impact of daily vitamin D_3_ supplementation on the risk of vitamin D deficiency with the interaction of rs2282679 in vitamin D binding protein gene (GC) among overweight and obese children and adolescents: A one-year randomized controlled trial. Front. Nutr..

[37] Tang Y., Huang Y., Luo L., Xu M., Deng D., Fang Z., Zhao X., Chen M. (2023). Level of 25-hydroxyvitamin D and vitamin D receptor in diabetic foot ulcer and factor associated with diabetic foot ulcers. Diabetol. Metab. Syndr..

[38] Song Z., Xiao C., Jia X., Luo C., Shi L., Xia R., Zhu J., Zhang S. (2021). Vitamin D/VDR Protects Against Diabetic Kidney Disease by Restoring Podocytes Autophagy. Diabetes Metab. Syndr. Obes..

[39] Wang B., Qian J.Y., Tang T.T., Lin L.L., Yu N., Guo H.L., Ni W.J., Lv L.L., Wen Y., Li Z.L. (2021). VDR/Atg3 Axis Regulates Slit Diaphragm to Tight Junction Transition via p62-Mediated Autophagy Pathway in Diabetic Nephropathy. Diabetes.

[40] Hou X., Xu F., Zhang C., Shuai J., Huang Z., Liang Y., Xu X. (2020). Dexmedetomidine exerts neuroprotective effects during high glucose-induced neural injury by inhibiting miR-125b. Biosci. Rep..

[41] Hu Y.J., Song C.S., Jiang N. (2022). Single nucleotide variations in the development of diabetic foot ulcer: A narrative review. World J. Diabetes.

[42] Zhao J., Zhang L.X., Wang Y.T., Li Y., Chen Md H.L. (2022). Genetic Polymorphisms and the Risk of Diabetic Foot: A Systematic Review and Meta-Analyses. Int. J. Low Extrem. Wounds.

[43] Soroush N., Radfar M., Hamidi A.K., Abdollahi M., Qorbani M., Razi F., Esfahani E.N., Amoli M.M. (2017). Vitamin D receptor gene FokI variant in diabetic foot ulcer and its relation with oxidative stress. Gene.

[44] Klashami Z.N., Ahrabi N.Z., Ahrabi Y.S., Hasanzad M., Asadi M., Amoli M.M. (2022). The vitamin D receptor gene variants, ApaI, TaqI, BsmI, and FokI in diabetic foot ulcer and their association with oxidative stress. Mol. Biol. Rep..

[45] Kurian S.J., Baral T., Benson R., Munisamy M., Saravu K., Rodrigues G.S., Sunil Krishna M., Shetty S., Kumar A., Miraj S.S. (2024). Association of vitamin D status and vitamin D receptor polymorphism in diabetic foot ulcer patients: A prospective observational study in a South-Indian tertiary healthcare facility. Int. Wound J..

[46] Gonzalez-Curiel I., Trujillo V., Montoya-Rosales A., Rincon K., Rivas-Calderon B., deHaro-Acosta J., Marin-Luevano P., Lozano-Lopez D., Enciso-Moreno J.A., Rivas-Santiago B. (2014). 1,25-dihydroxyvitamin D_3_ induces LL-37 and HBD-2 production in keratinocytes from diabetic foot ulcers promoting wound healing: An in vitro model. PLoS ONE.

[47] Jiang J., Zhang Y., Indra A.K., Ganguli-Indra G., Le M.N., Wang H., Hollins R.R., Reilly D.A., Carlson M.A., Gallo R.L. (2018). 1α,25-dihydroxyvitamin D_3_-eluting nanofibrous dressings induce endogenous antimicrobial peptide expression. Nanomedicine.

[48] Wu X., He W., Mu X., Liu Y., Deng J., Liu Y., Nie X. (2022). Macrophage polarization in diabetic wound healing. Burns Trauma.

[49] Zhang X., Zhao Y., Zhu X., Guo Y., Yang Y., Jiang Y., Liu B. (2019). Active vitamin D regulates macrophage M1/M2 phenotypes via the STAT-1-TREM-1 pathway in diabetic nephropathy. J. Cell. Physiol..

[50] Luo W.J., Dong X.W., Ye H., Zhao Q.S., Zhang Q.B., Guo W.Y., Liu H.W., Xu F. (2024). Vitamin D 1,25-Dihydroxyvitamin D_3_ reduces lipid accumulation in hepatocytes by inhibiting M1 macrophage polarization. World J. Gastrointest. Oncol..

[51] Wang Y., Wan L., Zhang Z., Li J., Qu M., Zhou Q. (2021). Topical calcitriol application promotes diabetic corneal wound healing and reinnervation through inhibiting NLRP3 inflammasome activation. Exp. Eye Res..

[52] Tang S., Tan J., Yang S., Li A., Liu J., Zhang W., Zhang H., Liu Y. (2024). Paricalcitol ameliorates diabetic nephropathy by promoting EETs and M2 macrophage polarization and inhibiting inflammation by regulating VDR/CYP2J2 axis. Faseb J..

[53] Hwang J., You H., Kwon D.H., Son Y., Lee G.Y., Han S.N. (2023). Transcriptome analysis of T cells from Ldlr(-/-) mice and effects of in vitro vitamin D treatment. J. Nutr. Biochem..

[54] Rodríguez-Carlos A., Trujillo V., Gonzalez-Curiel I., Marin-Luevano S., Torres-Juarez F., Santos-Mena A., Rivas-Santiago C., Enciso-Moreno J.A., Zaga-Clavellina V., Rivas-Santiago B. (2020). Host Defense Peptide RNase 7 Is Down-regulated in the Skin of Diabetic Patients with or without Chronic Ulcers, and its Expression is Altered with Metformin. Arch. Med. Res..

[55] Gottlieb C., Henrich M., Liu P.T., Yacoubian V., Wang J., Chun R., Adams J.S. (2023). High-Throughput CAMP Assay (HiTCA): A Novel Tool for Evaluating the Vitamin D-Dependent Antimicrobial Response. Nutrients.

[56] Benson R., Unnikrishnan M.K., Kurian S.J., Velladath S.U., Rodrigues G.S., Chandrashekar Hariharapura R., Muraleedharan A., Bangalore Venkateshiah D., Banerjee B., Mukhopadhyay C. (2023). Vitamin D attenuates biofilm-associated infections via immunomodulation and cathelicidin expression: A narrative review. Expert. Rev. Anti. Infect. Ther..

[57] Lowry M.B., Guo C., Zhang Y., Fantacone M.L., Logan I.E., Campbell Y., Zhang W., Le M., Indra A.K., Ganguli-Indra G. (2020). A mouse model for vitamin D-induced human cathelicidin antimicrobial peptide gene expression. J. Steroid Biochem. Mol. Biol..

[58] Tiwari S., Pratyush D.D., Gupta B., Dwivedi A., Chaudhary S., Rayicherla R.K., Gupta S.K., Singh S.K. (2013). Prevalence and severity of vitamin D deficiency in patients with diabetic foot infection. Br. J. Nutr..

[59] Tiwari S., Pratyush D.D., Gupta S.K., Singh S.K. (2014). Vitamin D deficiency is associated with inflammatory cytokine concentrations in patients with diabetic foot infection. Br. J. Nutr..

[60] Danny Darlington C.J., Kumar S.S., Jagdish S., Sridhar M.G. (2019). Evaluation of Serum Vitamin D Levels in Diabetic Foot Infections: A Cross-Sectional Study in a Tertiary Care Center in South India. Iran. J. Med. Sci..

[61] Greiller C.L., Suri R., Jolliffe D.A., Kebadze T., Hirsman A.G., Griffiths C.J., Johnston S.L., Martineau A.R. (2019). Vitamin D attenuates rhinovirus-induced expression of intercellular adhesion molecule-1 (ICAM-1) and platelet-activating factor receptor (PAFR) in respiratory epithelial cells. J. Steroid Biochem. Mol. Biol..

[62] Wang X., Meng L., Zhang J., Zou L., Jia Z., Han X., Zhao L., Song M., Zhang Z., Zong J. (2023). Identification of angiogenesis-related genes in diabetic foot ulcer using machine learning algorithms. Heliyon.

[63] Saber S., Abdelhady R., Elhemely M.A., Elmorsy E.A., Hamad R.S., Abdel-Reheim M.A., El-Kott A.F., AlShehri M.A., Morsy K., Negm S. (2024). Nanoscale Systems for Local Activation of Hypoxia-Inducible Factor-1 Alpha: A New Approach in Diabetic Wound Management. Int. J. Nanomed..

[64] Kim D.H., Meza C.A., Clarke H., Kim J.S., Hickner R.C. (2020). Vitamin D and Endothelial Function. Nutrients.

[65] Wang Y., Jiang L. (2021). Role of vitamin D-vitamin D receptor signaling on hyperoxia-induced bronchopulmonary dysplasia in neonatal rats. Pediatr. Pulmonol..

[66] Xu Q., Liu Z., Guo L., Liu R., Li R., Chu X., Yang J., Luo J., Chen F., Deng M. (2019). Hypoxia Mediates Runt-Related Transcription Factor 2 Expression via Induction of Vascular Endothelial Growth Factor in Periodontal Ligament Stem Cells. Mol. Cells.

[67] Arfian N., Kusuma M.H., Anggorowati N., Nugroho D.B., Jeffilano A., Suzuki Y., Ikeda K., Emoto N. (2018). Vitamin D upregulates endothelin-1, ETBR, eNOS mRNA expression and attenuates vascular remodelling and ischemia in kidney fibrosis model in mice. Physiol. Res..

[68] Khan A., Dawoud H., Malinski T. (2018). Nanomedical studies of the restoration of nitric oxide/peroxynitrite balance in dysfunctional endothelium by 1,25-dihydroxy vitamin D_3_—Clinical implications for cardiovascular diseases. Int. J. Nanomed..

[69] Wu M., Wu Y., Xu K., Lin L. (2021). Protective Effects of 1,25 Dihydroxyvitamin D_3_ against High-Glucose-Induced Damage in Human Umbilical Vein Endothelial Cells Involve Activation of Nrf2 Antioxidant Signaling. J. Vasc. Res..

[70] Miao D., Goltzman D. (2023). Mechanisms of action of vitamin D in delaying aging and preventing disease by inhibiting oxidative stress. Vitam. Horm..

[71] Li Y., Zhou Q., Pei C., Liu B., Li M., Fang L., Sun Y., Li Y., Meng S. (2016). Hyperglycemia and Advanced Glycation End Products Regulate miR-126 Expression in Endothelial Progenitor Cells. J. Vasc. Res..

[72] Chang M., Zhang B., Tian Y., Hu M., Zhang G., Di Z., Wang X., Liu Z., Gu N., Liu Y. (2017). AGEs Decreased SIRT3 Expression and SIRT3 Activation Protected AGEs-Induced EPCs’ Dysfunction and Strengthened Anti-oxidant Capacity. Inflammation.

[73] Xiong Y., Zhou F., Liu Y., Yi Z., Wang X., Wu Y., Gong P. (2021). 1α,25-Dihydroxyvitamin D_3_ promotes angiogenesis by alleviating AGEs-induced autophagy. Arch. Biochem. Biophys..

[74] Ye B., Weng Y., Lin S., Lin J., Huang Z., Huang W., Cai X. (2020). 1,25(OH)_2_D_3_ Strengthens the Vasculogenesis of Multipotent Mesenchymal Stromal Cells from Rat Bone Marrow by Regulating the PI3K/AKT Pathway. Drug Des. Devel. Ther..

[75] Liu Z., Sun H., Chen Y., He J., Zhu L., Yang B., Zhao W. (2024). High glucose-induced injury in human umbilical vein endothelial cells is alleviated by vitamin D supplementation through downregulation of TIPE1. Diabetol. Metab. Syndr..

[76] Sharifzadeh M., Esmaeili-Bandboni A., Emami M.R., Naeini F., Zarezadeh M., Javanbakht M.H. (2022). The effects of all trans retinoic acid, vitamin D_3_ and their combination on plasma levels of miRNA-125a-5p, miRNA-34a, and miRNA-126 in an experimental model of diabetes. Avicenna J. Phytomed.

[77] Veves A., Backonja M., Malik R.A. (2008). Painful diabetic neuropathy: Epidemiology, natural history, early diagnosis, and treatment options. Pain. Med..

[78] Eid S.A., Rumora A.E., Beirowski B., Bennett D.L., Hur J., Savelieff M.G., Feldman E.L. (2023). New perspectives in diabetic neuropathy. Neuron.

[79] Riaz S., Malcangio M., Miller M., Tomlinson D.R. (1999). A vitamin D_3_ derivative (CB1093) induces nerve growth factor and prevents neurotrophic deficits in streptozotocin-diabetic rats. Diabetologia.

[80] Lu X., Chen Z., Lu J., Watsky M.A. (2023). Effects of 1,25-Vitamin D_3_ and 24,25-Vitamin D_3_ on Corneal Nerve Regeneration in Diabetic Mice. Biomolecules.

[81] Dimova R., Tankova T., Chakarova N. (2017). Vitamin D in the Spectrum of Prediabetes and Cardiovascular Autonomic Dysfunction. J. Nutr..

[82] Long W., Fatehi M., Soni S., Panigrahi R., Philippaert K., Yu Y., Kelly R., Boonen B., Barr A., Golec D. (2020). Vitamin D is an endogenous partial agonist of the transient receptor potential vanilloid 1 channel. J. Physiol..

[83] Gündüz Başçıl S., Gölgeli A. (2022). Investigation of antinociceptive effects of vitamin D and EB1089 in rats. Agri.

[84] Robilotto G.L., Mohapatra D.P., Shepherd A.J., Mickle A.D. (2022). Role of Src kinase in regulating protein kinase C mediated phosphorylation of TRPV1. Eur. J. Pain.

[85] Britti E., Delaspre F., Sanz-Alcázar A., Medina-Carbonero M., Llovera M., Purroy R., Mincheva-Tasheva S., Tamarit J., Ros J. (2021). Calcitriol increases frataxin levels and restores mitochondrial function in cell models of Friedreich Ataxia. Biochem. J..

[86] Qiao J., Ma H., Chen M., Bai J. (2023). Vitamin D alleviates neuronal injury in cerebral ischemia-reperfusion via enhancing the Nrf2/HO-1 antioxidant pathway to counteract NLRP3-mediated pyroptosis. J. Neuropathol. Exp. Neurol..

[87] Fei S., Fan J., Cao J., Chen H., Wang X., Pan Q. (2024). Vitamin D deficiency increases the risk of diabetic peripheral neuropathy in elderly type 2 diabetes mellitus patients by predominantly increasing large-fiber lesions. Diabetes Res. Clin. Pract..

[88] Pang C., Yu H., Cai Y., Song M., Feng F., Gao L., Li K., Chen Y., Xie J., Cheng Y. (2023). Vitamin D and diabetic peripheral neuropathy: A multi-centre nerve conduction study among Chinese patients with type 2 diabetes. Diabetes Metab. Res. Rev..

[89] Alam U., Petropoulos I.N., Ponirakis G., Ferdousi M., Asghar O., Jeziorska M., Marshall A., Boulton A.J.M., Efron N., Malik R.A. (2021). Vitamin D deficiency is associated with painful diabetic neuropathy. Diabetes Metab. Res. Rev..

[90] Putz Z., Tordai D., Hajdú N., Vági O.E., Kempler M., Békeffy M., Körei A.E., Istenes I., Horváth V., Stoian A.P. (2022). Vitamin D in the Prevention and Treatment of Diabetic Neuropathy. Clin. Ther..

[91] Smart H., AlGhareeb A.M., Smart S.A. (2019). 25-Hydroxyvitamin D Deficiency: Impacting Deep-Wound Infection and Poor Healing Outcomes in Patients With Diabetes. Adv. Skin. Wound Care.

[92] Tang W., Chen L., Ma W., Liu G., Chen D., Wang C., Gao Y., Ran X. (2023). Association of vitamin D status with all-cause mortality and outcomes among Chinese individuals with diabetic foot ulcers. J. Diabetes Investig..

[93] Kinesya E., Santoso D., Gde Arya N., Putri Cintya E., Seriari Ambarini P., Kinesya B., Stephanie Kartjito M., Mannagalli Y. (2023). Vitamin D as adjuvant therapy for diabetic foot ulcers: Systematic review and meta-analysis approach. Clin. Nutr. ESPEN.

[94] Razzaghi R., Pourbagheri H., Momen-Heravi M., Bahmani F., Shadi J., Soleimani Z., Asemi Z. (2017). The effects of vitamin D supplementation on wound healing and metabolic status in patients with diabetic foot ulcer: A randomized, double-blind, placebo-controlled trial. J. Diabetes Complications.

[95] Kamble A., Ambad R., Padamwar M., Kakade A., Yeola M. (2020). To study the effect of oral vitamin D supplements on wound healing in patient with diabetic foot ulcer and its effect on lipid metabolism. Int. J. Res. Pharm. Sci..

[96] Mozaffari-Khosravi H., Haratian-Arab M., MoeinTavakkoli H., Nadjarzadeh A. (2017). Comparative effect of two different doses of vitamin D on diabetic foot ulcer and inflammatory indices among the type 2 diabetic patients a randomized clinical trial. Iran. J. Diabetes Obes..

[97] Halschou-Jensen P.M., Sauer J., Bouchelouche P., Fabrin J., Brorson S., Ohrt-Nissen S. (2021). Improved Healing of Diabetic Foot Ulcers After High-dose Vitamin D: A Randomized Double-blinded Clinical Trial. Int. J. Low Extrem. Wounds.

[98] Perna S. (2022). The enigma of vitamin D supplementation in aging with obesity. Minerva Gastroenterol..

[99] Shroff R., Wan M., Nagler E.V., Bakkaloglu S., Cozzolino M., Bacchetta J., Edefonti A., Stefanidis C.J., Vande Walle J., Ariceta G. (2017). Clinical practice recommendations for treatment with active vitamin D analogues in children with chronic kidney disease Stages 2-5 and on dialysis. Nephrol. Dial. Transplant..

[100] Cardoso M.M.A., Machado-Rugolo J., Lima S.A.M., Andrade L.G.M., Curado D.S.P., Ponce D. (2023). Cost-effectiveness analysis of intravenous paricalcitol vs. oral calcitriol in the treatment of hyperparathyroidism secondary to chronic kidney disease. J. Bras. Nefrol..

[101] Reichrath J., Saternus R., Vogt T. (2017). Challenge and perspective: The relevance of ultraviolet (UV) radiation and the vitamin D endocrine system (VDES) for psoriasis and other inflammatory skin diseases. Photochem. Photobiol. Sci..

[102] Gisondi P., Gracia-Cazaña T., Kurzen H., Galván J. (2024). Calcipotriol/Betamethasone Dipropionate for the Treatment of Psoriasis: Mechanism of Action and Evidence of Efficacy and Safety versus Topical Corticosteroids. J. Clin. Med..

[103] Lu X., Chen Z., Lu J., Watsky M. (2023). Effects of Topical 1,25 and 24,25 Vitamin D on Diabetic, Vitamin D Deficient and Vitamin D Receptor Knockout Mouse Corneal Wound Healing. Biomolecules.

[104] Wsoo M.A., Razak S.I.A., Bohari S.P.M., Shahir S., Salihu R., Kadir M.R.A., Nayan N.H.M. (2021). Vitamin D_3_-loaded electrospun cellulose acetate/polycaprolactone nanofibers: Characterization, in-vitro drug release and cytotoxicity studies. Int. J. Biol. Macromol..

[105] Ehterami A., Salehi M., Farzamfar S., Samadian H., Vaez A., Sahrapeyma H., Ghorbani S. (2020). A promising wound dressing based on alginate hydrogels containing vitamin D_3_ cross-linked by calcium carbonate/d-glucono-δ-lactone. Biomed. Eng. Lett..

[106] Kim D.S., Kim J.H., Baek S.W., Lee J.K., Park S.Y., Choi B., Kim T.H., Min K., Han D.K. (2022). Controlled vitamin D delivery with injectable hyaluronic acid-based hydrogel for restoration of tendinopathy. J. Tissue Eng..

[107] Kim C., Park S., Rho S.J., Kim Y.-R. (2025). Rice flour-based filled hydrogel: An effective vitamin D encapsulation system as influenced by rice flour variety. Food Sci. Biotechnol..

[108] Brahmbhatt A., NievesTorres E., Yang B., Edwards W.D., Roy Chaudhury P., Lee M.K., Kong H., Mukhopadhyay D., Kumar R., Misra S. (2014). The role of Iex-1 in the pathogenesis of venous neointimal hyperplasia associated with hemodialysis arteriovenous fistula. PLoS ONE.

[109] Cai C., Kilari S., Zhao C., Singh A.K., Simeon M.L., Misra A., Li Y., Takahashi E., Kumar R., Misra S. (2021). Adventitial delivery of nanoparticles encapsulated with 1α, 25-dihydroxyvitamin D_3_ attenuates restenosis in a murine angioplasty model. Sci. Rep..

[110] Macido A. (2018). Diabetic Foot Ulcers and Vitamin D Status: A Literature Review. SAGE Open Nurs..

[111] Greenhagen R.M., Frykberg R.G., Wukich D.K. (2019). Serum vitamin D and diabetic foot complications. Diabet. Foot Ankle.

[112] Kurian S.J., Miraj S.S., Benson R., Munisamy M., Saravu K., Rodrigues G.S., Rao M. (2021). Vitamin D Supplementation in Diabetic Foot Ulcers: A Current Perspective. Curr. Diabetes Rev..

[113] Liu L., Zhang F., Jamali M., Guimarães N.S., Radkhah N., Jamilian P., Wang Q. (2024). The role of vitamin D in diabetic foot ulcer; an umbrella review of meta-analyses. Front. Nutr..

